# Supplementary material for: Impacts of large-scale deployment of vertical bifacial photovoltaics on European electricity market dynamics
Source: Nat Commun. 2024 Aug 6;15:6681. doi: 10.1038/s41467-024-50762-7 (PMC11303785; doi:10.1038/s41467-024-50762-7)
Supplement: Supplementary file 2 — Description of Additional Supplementary Files DOCX [file 41467_2024_50762_MOESM2_ESM.docx]

**Description of Additional Supplementary Files**

File Name: Supplementary Data 1

Description: Gives the input data on Solar Irradiation (in W/sq.m) and the PV production data (in kWh/kWp) for 43 European countries for each hour of every month, and a summary of the average values. The datasets cover the South facing optimal inclination

File Name: Supplementary Data 2

Description: Gives the input data on Solar Irradiation (in W/sq.m) and the PV production data (in kWh/kWp) for 43 European countries for each hour of every month, and a summary of the average values. The datasets cover the East Facing Vertical PV.

File Name: Supplementary Data 3

Description: Gives the input data on Solar Irradiation (in W/sq.m) and the PV production data (in kWh/kWp) for 43 European countries for each hour of every month, and a summary of the average values. The datasets cover the West facing vertical PV.

File Name: Supplementary Data 4

Description: Gives the input data on Solar Irradiation (in W/sq.m) and the PV production data (in kWh/kWp) for 43 European countries for each hour of every month, and a summary of the average values. The datasets cover South facing vertical PV.

File Name: Supplementary Data 5

Description: Contains the input parameters and data used in the applied model for each scenario.

File Name: Supplementary Data 6

Description: Contains the output data of the applied model that are displayed in Figures 2 to 7:

• Figure 2 illustrates the calculated Total annualized costs for the wind and PV capacities combinations;

• Figure 3 displays the modelled technology capacity development levels for 2030 and for 2040 for three modelled scenarios and for the actual 2022 values;

• Figure 4 illustrates the impacts of reaching a mix of 50-50% optimal orientation and East-West facing bifacial PV on the future generation mix.

• Figure 5 covers eight specific impact areas in the future electricity sector for 2040: Total PV production (in GWh) Total curtailment (in GWh); Average baseload price (in €/MWh); Average PV market value, (in €/MWh); CO2 emissions (in kt); Operational cost (in bn€); Wind annualized investment cost, (in bn €); PV annualized investment cost (in bn€).

• Figure 6 provides a comparison country specific generation changes of various mixes of optimal and East-West facing PV modules for 15 selected representative EU countries.

• Figure 7 describes the wholesale price distribution in two scenarios for the selected EU countries.
